# Targets of statins intervention in LDL-C metabolism: Gut microbiota

**DOI:** 10.3389/fcvm.2022.972603

**Published:** 2022-09-08

**Authors:** ChangXin Sun, ZePing Wang, LanQing Hu, XiaoNan Zhang, JiYe Chen, ZongLiang Yu, LongTao Liu, Min Wu

**Affiliations:** ^1^Beijing University of Chinese Medicine, Beijing, China; ^2^Xiyuan Hospital, China Academy of Chinese Medical Sciences, Beijing, China; ^3^Guang'anmen Hospital, China Academy of Chinese Medical Sciences, Beijing, China

**Keywords:** gut microbiota, low density lipid cholesterol, cholesterol metabolism, statins, potential pathways

## Abstract

Increasing researches have considered gut microbiota as a new “metabolic organ,” which mediates the occurrence and development of metabolic diseases. In addition, the liver is an important organ of lipid metabolism, and abnormal lipid metabolism can cause the elevation of blood lipids. Among them, elevated low-density lipoprotein cholesterol (LDL-C) is related with ectopic lipid deposition and metabolic diseases, and statins are widely used to lower LDL-C. In recent years, the gut microbiota has been shown to mediate statins efficacy, both in animals and humans. The effect of statins on microbiota abundance has been deeply explored, and the pathways through which statins reduce the LDL-C levels by affecting the abundance of microbiota have gradually been explored. In this review, we discussed the interaction between gut microbiota and cholesterol metabolism, especially the cholesterol-lowering effect of statins mediated by gut microbiota, *via* AMPK-PPARγ-SREBP1C/2, FXR and PXR-related, and LPS-TLR4-Myd88 pathways, which may help to explain the individual differences in statins efficacy.

## Introduction

Blood lipids come from two sources. Exogenous fat is absorbed through the intestine and then decomposed into lipid particles, and endogenous lipids particles are generated in liver. All lipids particles can only be transported in the blood by binding to the corresponding apolipoprotein. Dyslipidemia mainly includes hypertriglyceridemia, low high-density lipoprotein cholesterol (HDL-C), high low-density lipoprotein cholesterol (LDL-C), and combined hyperlipidemia. In addition, high level of LDL-C can cause ectopic lipid deposition and associated metabolic diseases, such as non-alcoholic fatty liver disease (1), hypertension (2), and atherosclerosis (AS) (3). Among these, stroke and ischemic heart disease, which are AS-related diseases, are main causes of premature death among the Chinese population (4). Therefore, as the most effective drug against AS, statins remain an important choice especially for those whose LDL-C cannot be sufficiently reduced through modification of diet and living habits (5). However, elder patients with other comorbidities are more prone to the side effects of statins and face increased risks of musculoskeletal symptoms, diabetes, and hemorrhagic stroke (5, 6), which largely limits the use of statins.

Gut microbiota regulates several diseases, including liver diseases (7), neuropsychiatric disorders (8), and metabolic diseases, including type 2 diabetes, dyslipidemia, and obesity (9), mainly by regulating energy homeostasis, glucose metabolism, lipid metabolism, and other metabolic processes (10). As a critical part of the gut-liver axis, gut microbiota performs several functions in the metabolism of short-chain fatty acid (SCFA), bile acid (BA), trimethylamine-N-oxide (TMAO), and lipopolysaccharides (LPS), and has a significant impact on immunological, metabolic, structural, and neurological landscapes in the human body (11). Presently, the specific effects of lipid-lowering drugs, such as statins, on the gut microbiota remain unclear (12). This review elucidates how various statins affect the gut microbiota and LDL-C metabolism through the gut-liver axis to discover their potential therapeutic implications and explain why the efficacy of statins varies in different populations.

## Microbial effects on cholesterol metabolism

Gut microbiota, which is influenced by diet and living environment, is individually unique and highly related to cholesterol metabolism (13). Commonly, BAs and other active substances synthesized by the liver are secreted into the upper part of the small intestine, and affect the abundance of gut microbiota and microbiota-derived metabolites (MDMs) in jejunum, ileum and colon. Similarly, based on the mesenteric vein-portal vein system, metabolites in the gut are absorbed and transported directly to the liver through the gut mucosa and then transformed into metabolic raw materials and regulatory molecules. SCFA (14), secondary bile acid (SBA) (15), TMAO (16) and LPS (17) are main MDMs that can significantly regulate the cholesterol metabolism and even reduce LDL-C through the gut-liver axis. Thus, the gut microbiota and metabolites play key roles in regulating LDL-C levels.

The SCFAs, BAs, TMAO, and LPS are major gut microbiota metabolites that broadly affect the human cholesterol metabolism. SCFAs mainly include acetate, propionate, and butyrate, which are produced by the human gut microbiota through the fermentation of dietary fiber and amino acids obtained *via* hydrolysis using various pathways (18). The human gut microbiota includes the following genera: *Lactobacillus, Eubacterium, Faecalis, Bifidobacterium, Clostridium, Bacteroides* and *Butyrimionas* (19–22). *Lactobacillus* (23–25) and *Bifidobacterium* (26, 27). Increase in *Lactobacillus* and *Bifidobacterium* are positively correlated with the total SCFAs (28). Additionally, most members of the *Prevotellaceae, Clostridiaceae, Ruminococcaceae, Lactobacillaceae*, and *Lachnospiraceae* families are putative butyrate producers and own anti-inflammatory properties (29). *Clostridiaceae* and *Lachnospiraceae* are also acetate producers. Moreover, the *Erysipelotrichaceae* family is primarily responsible for the production of acetate and butyrate, based on metatranscriptome profiles (30). Studies have revealed that the *Coriobacteriaceae* family positively correlates with the serum LDL-C levels in mice fed with a high fat diet (HFD) (31).

BAs mainly exist as primary BAs, chenodeoxycholic acid (CDCA), and cholic acid (CA) before fermentation by gut microbiota, whereas the SBAs, deoxycholic acid (DCA), and lithocholic acid (LCA) are generated by *Clostridium* and some strains of *Lachnospiraceae* and *Peptostreptococcaceae* families in the gut; DCA and LCA are the main products (32). The *Clostridium* (33–35) and *Lachnospiraceae* (35) share the same trend of change with LDL-C levels. An experiment revealed that the percentage of Gram-positive bacteria, especially *Clostridium*, is significantly increased by HFD, which results in the production of DCA in the feces. Suppressing Gram-positive bacteria, including *Clostridium*, with antibiotics decreased the fecal DCA in this experiment (36). *Lactobacillus* can increase the relative proportion of intestinal *Clostridium* and the production of SBAs (DCA and LCA) in rats fed in a high-cholesterol diet (37). Moreover, *Lactobacillus* decreased the absorption of BAs and increased the excretion of BAs in feces (38). A significant correlation exists between the increase in *Bifidobacterium* and the decrease in fecal LCA (39). Low abundance of *Bacteroidetes* and high abundance of *Erysipelotrichaceae* indicate excessive SBAs in feces (40).

Methanogenic bacteria have been reported to deplete both trimethylamine (TMA) and TMAO (41, 42), and a randomized controlled trial showed that probiotic supplementation (such as *Lactobacillus* and *Bifidobacterium*) significantly increased the TMAO levels (43). Naghipour et al. revealed the production of TMA is related to three biochemical pathways. Based on genetic testing, comparing the genes involved in the biochemical pathways, it has been confirmed that the *Bacteroides* lack the related genes, whereas these genes exist in the *Firmicutes, Proteobacteria* and *Actinobacteria* phyla (44). Among them, reduction in *Firmicutes* (45), *Proteobacteria* and *Actinobacteria* (46) is often accompanied by reduction in the LDL-C levels. Falony et al. (47) suggested that *Bacteroidetes* did not produce TMA, and Wang et al. (48) observed that *Bacteroides* were negatively correlated with the plasma TMAO levels. However, a previous research revealed that TMA and TMAO levels were associated with an increased *Firmicutes*/*Bacteroidetes* ratio (49), which was consistent with the above findings.

LPS is specific to Gram-negative bacteria. At the phylum level, *Bacteroidetes* and *Proteobacteria* are the two most dominant Gram-negative bacteria in the gut microbiota. Although the relative abundance of *Bacteroidetes* decreased significantly after the HFD intervention, the relative abundance of *Proteobacteria* was observed to increase (50). In addition, the absolute abundance of the gut microbiota doubled by HFD intervention (51), which indicated that although the relative abundance of *Bacteroidetes* was reduced, the total number may not have changed significantly. We observed that SCFAs, BAS, TMAO, and LPS are related to the ability of the gut microbiota to regulate the LDL-C levels *via* metabolites (Table 1). However, correlative research reports are insufficient, and long-term studies are still needed to determine the impact of gut microbiota on SCFAs, BAs, TMAO, and LPS.

**Table 1:** Relationship between different microbiota and its metabolites.

**Authors**	**Gut microbiota relationship**	**Between gut and metabolites microbiota**	**References**
Lee et al.	Bifidobacterium, Clostridium, Faecalibacterium and Lactobacillus	Generate acetate, propionate, butyrate and others	(19)
Chang et al.	Blautia, Eubacterium, Collinsella and Subdoligranulum	Generate butyrate, valerate	(20)
Zhou et al.	Faecalibacterium	Generate butyrate	(21)
Kim et al.	Bacteroides, Butyricimonas, and Mucispirillum	Generate acetate, propionate, butyrate and others	(22)
Zhu et al.	Lactobacillus and Bifidobacterium	Generate Total SCFA	(28)
Esquivel-Elizondo et al.	Prevotellaceae, Clostridiaceae, Lactobacillaceae, Ruminococcaceae, and Lachnospiraceae	Generate butyrate	(29)
Hugenholtz et al.	Erysipelotrichaceae	Generate acetate and butyrate	(30)
Wang et al.	Clostridium, Lachnospiraceae and Peptostreptococcaceae	Generate DCA and LCA	(32)
Wang et al.	Clostridiumt	Generate DCA	(36)
Hu et al.	Clostridium	Generate DCA and LCA	(37)
Palaniyandi et al.	Lactobacillus	Increase Ba excretion	(38)
Okazaki et al.	Bifidobacterium	Decrease LCA	(39)
Kamp et al.	Bacteroidetesand, Erysipelotrichaceae	Positive correlation with SBAs	(40)
Brugère et al.	Methanogenic bacteria	Consumption of TMA and TMAO	(41)
Dridi et al.	Methanogenic bacteria	Consumption of TMA and TMAO	(42)
Chen et al.	Lactobacillus and Bifidobacterium	Decrease TMAO increase	(43)
Naghipour et al.	Firmicutes, Proteobacteria and Actinobacteria	Positive correlation with TMA level	(44)
Falony et al.	Bacteroidetes	Generate no TMA	(47)
Wang et al.	Bacteroides	Negative correlation with TMAO level	(48)
Cho et al.	Firmicutes / Bacteroidetes ratio	Positive correlation with TMA and TMAO	(49)

## Points of contact between gut microbiota and cholesterol metabolism

### SCFAs

Different SCFAs play different roles in the body; acetate's concentration is the highest in the blood, propionate helps in liver gluconeogenesis, and butyrate is the main energy source for the colon cells (52). Earlier experiments have demonstrated a relationship between SCFAs and LDL-C (53, 54). In addition, SCFAs significantly reduced the cholesterol levels in the plasma and liver of male rats (55), and the proliferation of bacteria that produce SCFAs can effectively reduce the blood cholesterol in hamsters with hypercholesterolemia (56). A HFD reduced the formation of SCFAs, whereas supplementing fermentable dietary fiber affected the SCFAs and reduced the plasma cholesterol levels (57).

A Previous research revealed that oral acetate (58, 59), propionate (60), and butyrate supplementation (61, 62) can improve the expression of lipid metabolism genes in mice with a HFD. Examples include the upregulation of cyclic adenosine monophosphate-responsive element binding protein-regulated transcription coactivator (CRTC) 2 and phosphorylated acetyl-CoA carboxylase (p-ACC), or downregulation of peroxisome proliferator-activated receptor (PPAR) γ, acetyl-CoA carboxylase (ACC), and sterol regulatory element binding protein 1 (SREBP1) (63). Among these, PPARγ is considered as the central medium for SCFA to play a beneficial role (64). The knockout of PPARγ in the liver completely abolished the effect of SCFAs, indicating that SCFAs act through related pathways in the liver. SCFAs acetate, propionate, and butyrate were the main pathways that inhibited PPARγ expression and activated the AMP-activated protein kinase (AMPK) pathway in this experiment (64). Activating the AMPK signaling pathway inhibited the expression of SREBP1c and SREBP2 directly (65). The SREBP plays a role in activating ACC and 3-hydroxy-3-methylglutaryl-CoA reductase (HMGCR) (66), among which HMGCR is the key enzyme for cholesterol synthesis. Thus, a decrease in SREBP expression is often accompanied by a decrease in plasma lipoprotein and cholesterol levels (67). Suppressing the AMPK-SREBP signaling pathway regulates cholesterol metabolism and prevents high LDL-C levels, which is beneficial for lowering LDL-C levels in serum and reducing liver lipid accumulation (66).

Among the free fatty acid receptors (FFAR), FFAR 2 and 3 are generally considered as SCFA receptors; however, studies have revealed that propionate and butyrate are not significantly associated with FFAR 3 (68). Acetate, propionate, and butyrate can activate FFAR 2 in the intestine and promote the secretion and intestinal peristalsis of peptide YY (PYY) and glucagon-like peptide 1 (GLP-1). Further studies have shown that HFD can reduce the expression of GLP-1 receptors in the liver, while supplementation with butyrate, a GLP-1 sensitizer, upregulates the levels of GLP-1 receptors in the liver, which stimulates AMPK phosphorylation (69). In general, the SCFAs-FFAR 2 signaling pathway is beneficial for improving cholesterol metabolism and reducing LDL-C levels; however, there is no current literature to explain the relationship between GLP-1 and the AMPK signaling pathway.

Studies have discovered that increased SCFAs induce the upregulation of cytochrome p-450 enzyme cholesterol 7α-hydroxylase (CYP7A1) (70) and 27α-hydroxylase (CYP27A1) (71) in the liver. Upregulation of CYP7A1 promotes cholesterol converted into BAs (72), thereby increasing cholesterol consumption and lowering LDL-C levels. Dietary acetate supplementation does not increase CYP7A1 mRNA levels (59), but increased propionate (53) and butyrate (54) levels can lead to the upregulation of CYP7A1, promote cholesterol catabolism, and reduce plasma LDL-C levels. Therefore, CYP7A1 is also a pathway for SCFAs to regulate cholesterol metabolism and LDL-C levels. In summary, SCFAs are closely related to the regulation of LDL-C via AMPK, FFAR 2, and CYP7A1-related signaling pathways, which are the main signaling pathways through which SCFAs regulate lipid metabolism and improve high LDL-C levels in the liver and intestine.

### Bile acids

BAs are important components of bile and are stored and concentrated in the gallbladder. BAs flow into the intestine under the control of filling and emptying of the gallbladder. Approximately 5% of BAs cannot be absorbed by hepatocytes through the hepatoenteric circulation and are excreted back into the bile (73). The liver maintains the stability of the BA pool by synthesizing BAs, which is the main catabolic pathway that consumes cholesterol in the body and is the only quantitative significant cholesterol catabolic mechanism (74). The nuclear receptor farnesoid X receptor (FXR) and cell surface receptor G protein-coupled bile acid receptor 5 (TGR5) are the key receptors required by Bas in maintaining systemic cholesterol levels and energy metabolism. The FXR and TGR5 signaling pathways regulate the liver BA synthesis and intestinal BA excretion *via* the FXR activation of TGR5 in the enterocytes (75).

CYP7A1 and CYP27A1 are involved in the first step of the convert of cholesterol into BAs (76); however, CYP7A1 is also a rate-limiting enzyme in the entire conversion process. CYP7A1 is simultaneously inhibited by an FXR-small heterodimer partner (SHP), which is the downstream target of FXR in the liver, whereas FXR in enterocytes inhibits CYP7A1 activation in hepatocytes by secreting fibroblast growth factor 19 (FGF19) and binding to fibroblast growth factor receptor 4 (FGFR4) on the hepatocyte membrane (77). The expression level of FGF19 was increased 16-fold in the ileum of rabbits fed in a high-cholesterol diet, and CYP7A1 decreased by 75% in the liver (78). A study by Jones revealed that the total cholesterol concentration in the liver after CYP7A1 knockout in mice was significantly higher than that in non-knockout mice (79). CDCA, CA, DCA, and LCA are all FXR agonists that can activate the FXR-CYP7A1 signaling pathway (80), which inhibits cholesterol consumption and causes abnormal cholesterol levels. Animal studies have confirmed that dietary supplementation with *Lactobacillus* can reduce the relative abundance of *Clostridium*, LDL-C/HDL-C, and the production of DCA and LCA in the intestines of rats that were fed in a high-cholesterol diet (37). In summary, changing *Clostridium* and *Lactobacillus* can regulate the production of LCA, DCA, and cholesterol consumption.

TGR5 can be activated by CDCA, CA, DCA, and LCA to promote the secretion and release of PYY and GLP-1 (81). Compared with normal mice, the expression levels of CYP7A1 and CYP27A1 increased in mice whose the TGR 5 gene was knocked out, indicating that TGR5 is involved in inhibiting the expression of CYP7A1 and CYP27A1 (82). However, there were also differences in the lipid-lowering effects of TGR5 between the female and male mice. LDL-C levels reduced significantly only in female mice whose TGR5 gene was knocked out.

Pregnane X receptor (PXR) is activated by LCA hepatoxic BAs to prevent cholestatic liver disease; however, it is not affected by CDCA, CA, or DCA (83). In addition, PXR can inhibit CYP7A1 transcription through SHP, which is a key activator of the activation of CYP7A1 gene in the hepatocytes (84). Additionally, PXR activation leads to the upregulation of SREBP2 and HMGCR and broadly induces cholesterol synthesis both in mice experiments and clinical researches (85). Kim et al. (86) discovered that PXR was related to a decrease in *Lactobacillus* and *Bifidobacterium* levels and an increase in the ratio of *Firmicutes*/*Bacteroides*. As a part of the gut-liver axis, BAs regulate the cholestatic metabolism *via* a multi-pathway process involving FXR, TGR5, and PXR, which are closely related to the LDL-C levels.

### Trimethylamine-N-oxide

TMA, which forms TMAO in the liver, is generated by the action of the gut microbiota by using dietary precursors such as choline, choline-containing compounds, betaine, and L-carnitine (87). Among these, choline is closely related to high TG and low HDL-C levels (88). Part of the TMA produced by the gut microbiota is fermented to produce methane by the gut microbiota, and the rest is absorbed into the liver (41).

TMAO is formed by flavin monooxygenase (FMO), which is expressed in the liver. Among the five members of the FMO family, only FMO1 and FMO3 can oxidize TMA to TMAO in the liver, especially FMO3 (89). FMO3 significantly decreases the expression of genes involved in *de novo* lipogenesis. In high-cholesterol diet mice, FMO3 knockout can affect the biliary lipid secretion and slow down cholesterol absorption in the intestine (90). Studies have revealed that the excretion of BAs indirectly induced by TMAO is a potential target for cholesterol-lowering treatments and is an independent risk factor. TMAO could cause an increase in serum LDL-C concentration and a decrease in the relative abundance of CYP7A1 in the hepatocytes; however, there was no significant change in the relative abundance of CYP27A1 (91–93). In addition, the expression of FXR (encoded by Nr1h4) and SHP (encoded by Nr0b2) were significantly upregulated by TMAO, which indicated that the FXR-SHN pathway is regulated by TMAO *via* CYP7A1 and cholesterol consumption (93). In mice not interfered with the gut microbiota, dietary supplementation with TMAO or precursors of TMA reduced *in vivo* reverse cholesterol transport (94). Antibiotics can reduce the production of TMAO by inhibiting the gut bacteria associated with TMAO (95). Moreover, further research has shown that oral broad-spectrum antibiotics can completely offset the effects of supplementing TMA precursors and reversing cholesterol transport (94).

### LPS

LPS, which is composed of lipids and polysaccharides, is a unique chemical component of the outer wall of Gram-negative bacteria. Generally, LPS is difficult to shed from the cell wall and sloughs off when bacteria die. LPS can enter the circulatory system after translocating by disrupting the intestinal epithelial barrier and increasing epithelial permeability.

In a meta-analysis of the gut microbiota and inflammatory markers, modulation of the gut microbiota reduced the LPS levels by 22–28% (96). Another meta-analysis showed that the translocation of LPS was directly related to the concentration of LDL-C, which was more pronounced in smokers or obese individuals (97, 98). LPS promotes hepatic proprotein convertase subtilisin/kexin-9 (PCSK9) synthesis that suppresses cholesterol consumption by binding to LDL-C receptors on cell membranes (17, 99). The mechanism by which LPS increases the synthesis of PCSK9 in the liver may be related to the phosphatidylinositol-3-kinase (PI3K)-Akt signaling pathway; further studies are required in this regard (100).

In few studies, the HMGCR mRNA levels in the liver increased after 4h of LPS treatment, which increased the hepatic cholesterol synthesis and serum cholesterol levels (101). *In vitro* studies by Ye et al. revealed that exposure to LPS resulted in the overexpression of SREBP2 and SREBP cleavage-activating protein (SCAP) (102). SCAP is required to activate SREBP1/2 in the hepatocytes, and the knockout of SCAP negatively regulated SREBP1/2 expression and cholesterol synthesis (103). Studies have demonstrated that LPS-induced inflammatory response leads to the downregulation of FXR, SHP, and CYP7A1, resulting in downregulation of LDL-C clearance and cholesterol consumption in the long term (104, 105). In addition, LPS inhibited the expression of liver receptor homolog (LRH) that can positively regulate CYP7A1, which may explain the relationship between CYP7A1, FXR and SHP (105).

Toll-like receptor 4 (TLR4) is considered as a potential receptor for LPS, and activates by an adaptor protein, such as myeloid differentiation factor 88 (Myd88). Prenatal exposure of mice to LPS (intraperitoneal injection) resulted in significantly elevated serum LDL-C levels and high LDL-C levels in the offspring. Cao et al. (2019) suggested more pronounce serum LDL-C changes in TLR2 knockout offspring mice and indicated that this may be due to a compensatory increase in TLR4 caused by TLR2 deficiency, manifested by an increased expression of TLR4 and Myd88 overactivity. Thus, the presence of LPS led to elevated serum LDL-C levels by activating TLR4 (106). Studies have discovered that the TLR4-Myd88 signaling pathway is related to lipid metabolism, as shown by the fact that the lipid-lowering effects of multiple lipid-lowering drugs are eliminated in Myd88 knockout mice (107). Elevated LPS in hepatocytes promoted the expression of SREBP2 by activating TLR4-Myd88 and the relative inflammatory response pathway (JNK/c-Jun) (108, 109).

## Statins-related potential pathways regulating cholesterol metabolism

### Statins and gut microbiota

Statins are well known as the most commonly used cholesterol-lowering drugs, which can directly reduce plasma cholesterol levels, especially LDL-C (110), by inhibiting cholesterol synthesis and HMGCR. The cholesterol-lowering effect of statins is closely related to the gut microbiota and can be impaired for the imbalance if gut microbiota (12, 51). In addition, different statins, such as atorvastatin, simvastatin, and rosuvastatin, have different effects on different microbiota (111) and thereby affect MDM (Table 2).

**Table 2:** The effect of some statins on the gut microbiota.

	**Gut microbiota**	**Changes**	**References**
Atorvastatin	*Lactobacillus, Eubacterium*, and *Faecalibacterium, Bifidobacterium* genus	More	(112)
	*Clostridium* genus	Less	(112)
Simvastatin	*Bacteroidetes* phylum, *Ruminococcaceae* and *Lactobacillus* genus	More	(113, 114)
	*Firmicutes* phylum	Less	(114)
Rosuvastatin	*Firmicutes* / *Bacteroidetes* ratio	Less	(22)
	*Bacteroides* and *Butyricimonas*	More	(22)
	*Firmicutes, Fusobacterium and Proteobacteria* phylum, *Ruminococcaceae, Lachnospiraceae, Clostridiaceae, Coriobacteriaceae, Erysipelotrichaceae, Akkermansiaceae* family and *Akkermansia*	Less	(115, 116)
	*Bacteroidaceae, Lachnospiraceae* and *Erysipelotrichaceae* families	More	(115, 116)

Compared to HFD mice that received atorvastatin, LDL-C levels were significantly higher in HFD mice that received atorvastatin and antibiotics. In this experiment, antibiotic-treated mice and mice without antibiotics showed different responses to statins, and LDL-lowering effect varied. LDL-C was lower in HFD mice that only received atorvastatin, whereas the results of HFD mice treated with atorvastatin and antibiotics were insignificant compared to untreated HFD mice (51). The diversity of gut microbiota changes after statin treatment (12), and is possibly related to a decrease in the incidence of gut microbiota imbalance (117). Sun et al. have revealed that hyperlipidemic patients with atorvastatin sensitivity manifested a higher diversity of gut microbiota, such as greater number of *Lactobacillus, Eubacterium, Faecalibacterium, Bifidobacterium* and fewer *Clostridium* genera (112).

Animal experiments have confirmed that the effect of simvastatin in reducing of serum LDL-C is affected by the gut microbiota. In a control experiment of simvastatin and antibiotics combined with simvastatin in HFD mice, serum LDL-C levels were significantly reduced in HFD mice that were treated with simvastatin, whereas serum LDL-C levels were higher in mice that were treated with antibiotics and simvastatin (118). A previous study showed that simvastatin changed the composition of gut microbiota in rats fed with HFD and caused an elevation in *Ruminococcaceae* and *Lactobacillus* levels (113). Another study showed that the relative abundance of *Firmicutes* and the portion of *Ruminococcaceae* decreased, while *Bacteroidetes* increased when followed simvastatin therapy (114).

**Figure 1:**
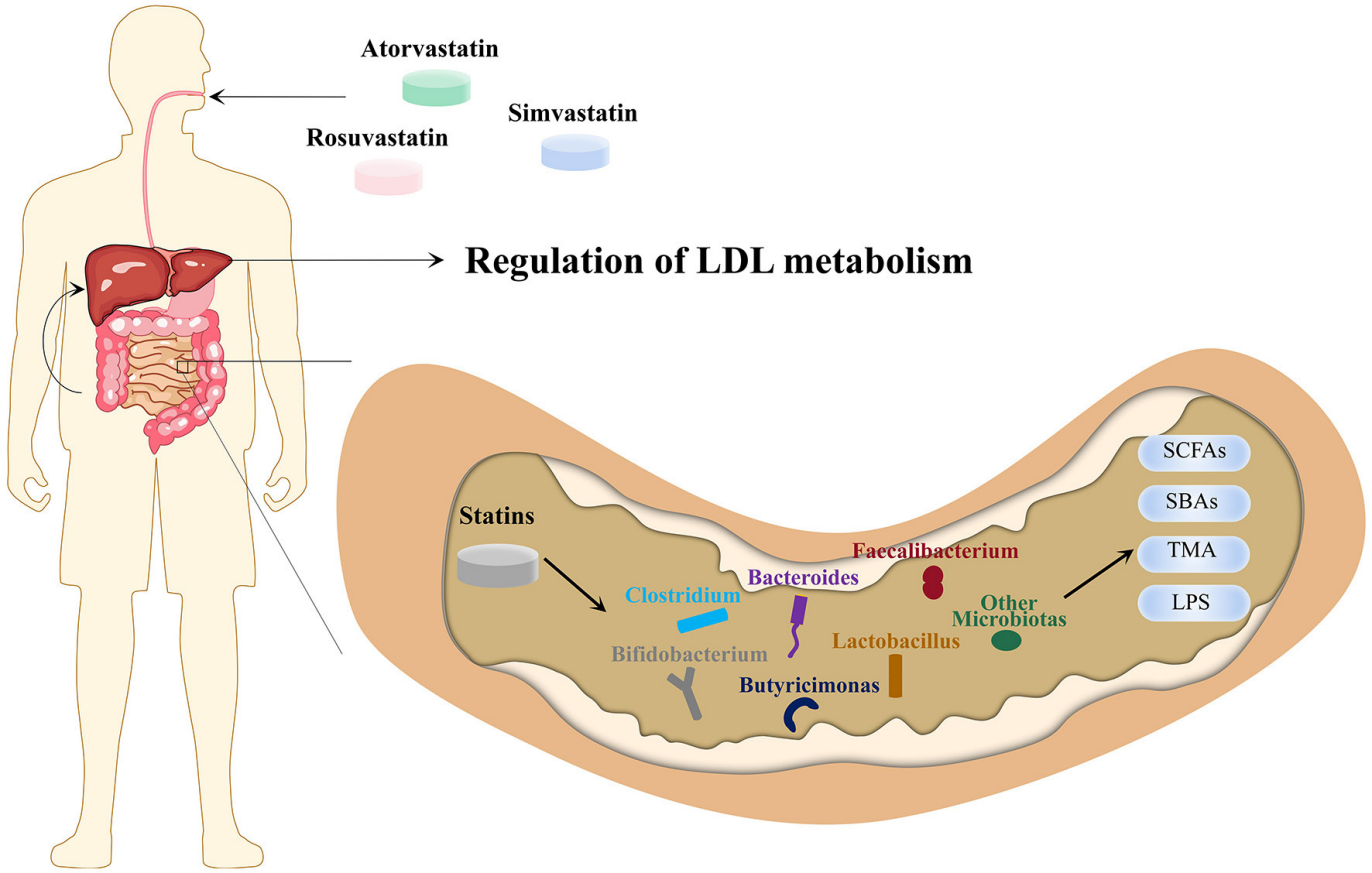
Microbiota-driven, LDL-lowering effect of statins. Atorvastatin, simvastatin and rosuvastatin enter the intestine *via* the upper gastrointestinal tract through oral administration and affect the absolute and relative abundance of gut microbiota, which in turn affects SCFAs, SBAs, TMA, LPS and other MDMs. The MDMs are transported to the liver through the portal venous system, ultimately achieving the purpose of regulating cholesterol metabolism and LDL-C levels. LPS, lipopolysaccharides; MDMs, microbiota-derived metabolites; LDL-C, low-density lipoprotein cholesterol; SBAs, secondary bile acid; SCFAs, short-chain fatty acid; TMA, trimethylamine.

Nolan et al. have suggested that the intervention of rosuvastatin in mice with HFD resulted in a significant increase in the *Lachnospiraceae* and *Erysipelotrichaceae* families and a significant decrease in *Proteobacteria* phylum, *Coriobacteriaceae, Akkermansiaceae* family, and *Akkermansia* (115). However, a clinical study revealed that the *Firmicutes* and *Fusobacterium* phyla and *Ruminococcaceae, Clostridiaceae, Lachnospiraceae, Coriobacteriaceae* and *Erysipelotrichaceae* families were negatively correlated with the LDL-lowering effect of rosuvastatin, while the *Bacteroidaceae* family had a positive correlation (116). The results of *Lachnospiraceae* and *Erysipelotrichaceae* families were opposite in the two experiments, and the changes in the rest of the gut microbiota in the two experiments were consistent. The application of rosuvastatin reduced the *Firmicutes*/*Bacteroidetes* ratio and increased the abundance of *Bacteroides* and *Butyricimonas* simultaneously (22). We found that statins can regulate cholesterol metabolism through MDM, and the major gut microbiota affected by statins (Figure 1).

### AMPK-PPARγ-SREBP1C/2

Regarding SCFA, there are more gut microbiota that produce SCFA in patients with hyperlipidemia who are sensitive to atorvastatin (112). The application of atorvastatin reduced the *Firmicutes*/*Bacteroidetes* ratio to a level close to that of normal mice (22), among which the abundance of butyrate-producing *Firmicutes* (such as the *Faecalibacterium*) had been increased by statins (119). Similarly butyrate-producing *Bacteroidetes* (including the *Bacteroides* and *Butyricimonas*) that were treated with atorvastatin and simvastatin also increased (22). However, Khan et al. observed that patients with high cholesterol levels had significantly higher levels of *Firmicutes* than those in healthy patients. The increase in the proportion of *Faecalibacterium* and *Bifidobacterium* in patients with high cholesterol treated with atorvastatin was consistent with the results of previous studies (120).

Hu et al. observed that the PPARγ and HMGCR protein expression decreased in the liver of HFD rats after atorvastatin intervention (121). The expression of HMGCR in mice treated with antibiotics was twice as high as that in mice without any special intervention (64), suggesting that HMGCR expression is partly influenced by the gut microbiota. The SREBP family is activated in low-cholesterol situation, in which SREBP2 plays a major role (102, 122). Therefore, it is challenging to observe changes in SREBP without being affected by cholesterol. Zimmermann et al. discovered that atorvastatin intervention increased the average levels of SREBP2 expression in the liver of HFD mice, and the effect of atorvastatin was reversed in antibiotic-treated HFD mice (51). This suggests that antibiotics attenuate the SREBP2 response to a low-cholesterol state by eliminating the positive effect of gut microbiota-mediated lipid-lowering action of atorvastatin.

In another experiment, the feces of HFD mice tended to have less acetate and butyrate and more propionate. Acetate and propionate levels increased significantly after simvastatin intervention, whereas butyrate levels did not change significantly. Similarly, the expression of HMGCR and SREBP1c were lower after simvastatin intervention in the liver and were closer to the levels in normal rats (113). Gu et al. also found that lipid metabolism-related genes SREBP1c and ACC1 in the liver were reduced after simvastatin treatment (123). SREBP and PCSK9 are closely related. Both SREBP1c and SREBP2 in fasting mice promoted the expression of hepatic PCSK9, which elevated serum PCSK9 levels (124). Thus, the lipid-lowering effect of simvastatin mediated by gut microbiota may be related to the reduction in SREBP1c levels.

In brief, atorvastatin can significantly regulate the serum LDL-C level in model animals by regulating the PPARγ signaling pathway and HMGCR gene expression.

### FXR and PXR signaling pathways

Atorvastatin treatment of hypercholesterolemic patients can selectively regulate the relative abundance of several functionally dominant gut microbiota. *Clostridium* is an important microbiota involved in producing SBAs and increasing SBA-mediated dyslipidemia induced by HFD. Reducing *Clostridium* can inhibit the production of SBAs, prevent CYP7A1 from being inhibited by FXR and PXR, and ultimately increase cholesterol consumption. Statins can be also used for *Clostridium* infections in the intestine (27). Moreover, the relative abundance of *Bifidobacterium*, which can reduce LCA, was decreased in hypercholesterolemic patients (120).

A previous study showed that the CYP7A1 induction by atorvastatin appears to be due to suppressed FXR signaling in the liver and intestine (125). Simvastatin promoted CYP7A1 expression in the liver of HFD mice; however, the effect of simvastatin was impaired by antibiotics. In addition, simvastatin significantly reduced serum CDCA and DCA levels in HFD mice but had no significant effect on CA and LCA levels. Antibiotics can cause an increase in CDCA and a decrease in LCA, which may be related to the unbalanced gut microbiota in the production of LCA (118). In experiments with the combined intervention of atorvastatin and antibiotics, average levels of hepatic FXR expression in control mice, HFD mice, and atorvastatin-treated mice were lower than in mice given the same intervention and antibiotics (51). This confirms the influence of gut microbiota on the lipid-lowering effect of statins. FXR-SHP-CYP7A1 signaling pathway is one of the lipid-lowering pathways of atorvastatin. Moreover, FXR can activate PXR through PPARα (126). Li et al. observed that reducing secondary BAs such as DCA and LCA can regulate cholesterol metabolism by inhibiting the FXR-SHP pathway and reducing the expression of SREBP1c through the FXR-PXR pathway (127).

Compared with HFD rats, simvastatin could intervention resulted in a significant decrease in LDL-C levels and a significant increase in total BAs excretion, which led to the promotion of cholesterol consumption in rats that were not fed an HFD. Zhang et al. revealed that simvastatin intervention could antagonize the inhibitory effect of HFD on the expression of CYP7A1 in the liver tissue of rats (113). In an experiment investigating PXR and BAs excretion, administration of atorvastatin lowered BAs levels in the intestine, but was of no use in PXR knockout mice (128). The PXR-SHP-CYP7A1 signaling pathway showed a new potential in increasing BA synthesis and promoting cholesterol consumption. In addition, Catry et al. discovered that simvastatin increased the expression of SREBP2 in the intestinal tract of mice and indicated that enterocyte SREBP2 overexpression was related to the expression of cholesterol excretion genes (129). This may explain how simvastatin increase the excretion of BAs.

### LPS-TLR4-Myd88

Gram-positive *Firmicutes* bacteria and Gram-negative *Bacteroidetes* bacteria account for 80% of the normal intestinal tract microbiota, and can exceed to 90% through an HFD (22). A previous study showed that the ileum was injured after exposure to low doses of LPS to the small intestine (130). LPS disrupted intestinal intercellular tight junction through a TLR4-dependent intracellular mechanism, resulting in increased intestinal permeability (131). However, we are yet to determine whether LPS damage to the gut affects the FXR and PXR pathways in enterocytes, thereby affecting the regulation of cholesterol by the gut microbiota. We observed that LPS translocation causes abnormal cholesterol metabolism and elevated serum LDL-C levels *via* TLR4-Myd88 in hepatocytes (106–109), which can be prevented by statins. Moreover, TLR4-Myd88 can activate both ERK and JNK signaling pathways in mice hepatocytes, thereby inhibiting CYP7A1 expression (132). ERK and JNK were important components of mitogen-activated protein kinases (MAPK) signaling pathway and were closely related to glucose and lipid metabolism (133). Repression of CYP7A1 by ERK and JNK is abolished when the Myd88 is disappeared in mice (132).

**Figure 2:**
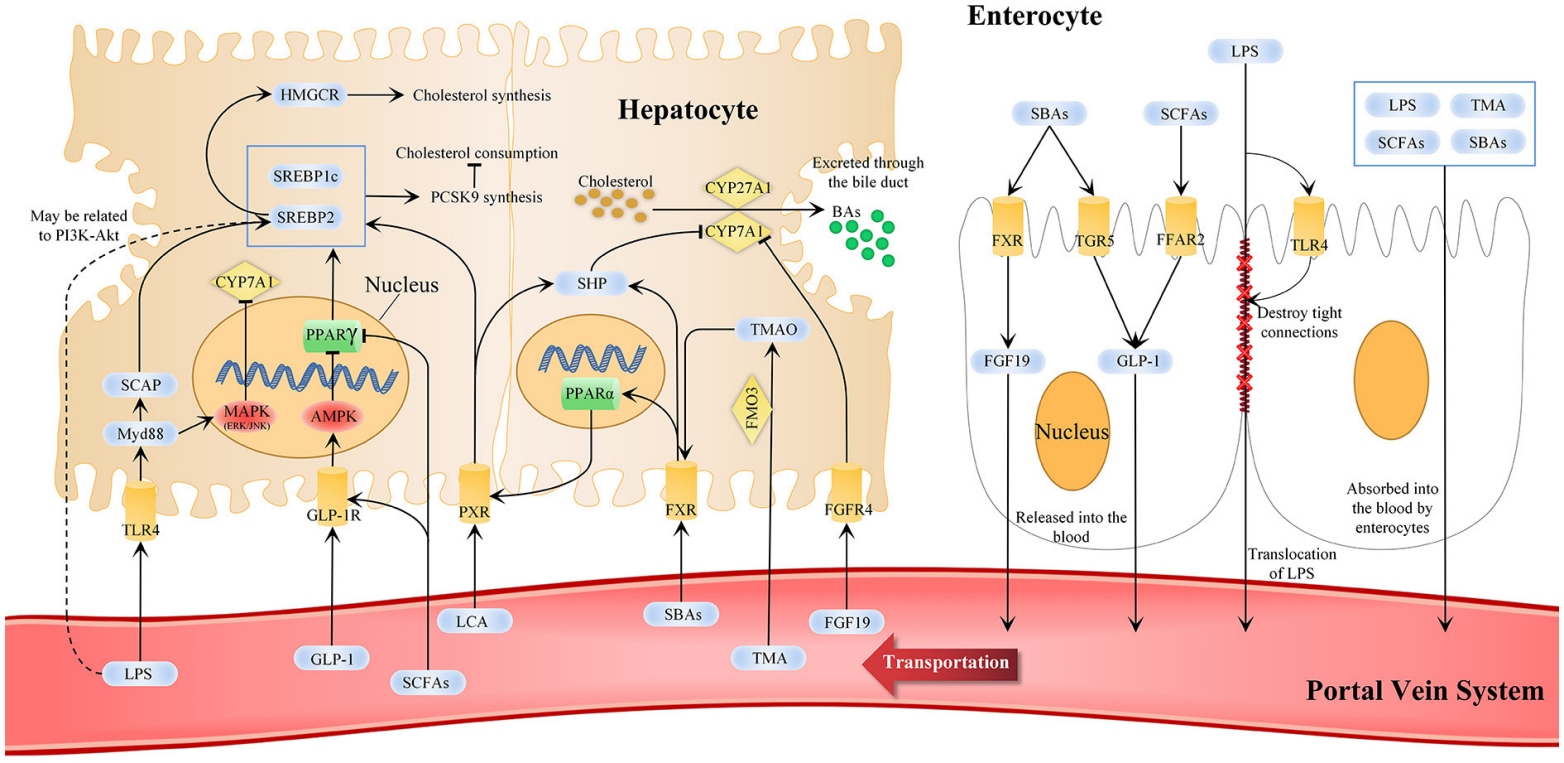
Statins-related microbiota metabolism and pathways in cholesterol metabolisms. In cholesterol metabolism, changes in gut microbiota composition and its metabolites are markers for regulating the gut-liver axis and can reflect the effect of statins. The liver-gut axis can be used as one of the ways statins affect cholesterol metabolism and can explain the why the efficacy of statins differs individually. SCFAs, SBAs, TMA, LPS, and other MDMs are changed after statins administration. SCFAs and SBAs can activate enterocytes to secrete FGF19 and GLP-1 into the blood, and others are absorbed by enterocytes into the blood circulation and finally absorbed in the liver through the portal vein system. This affects the AMPK-PPARγ-SREBP1C/2, FXR and TGR5 signaling pathways and LPS-TLR4-Myd88 and PI3K-Akt in hepatocytes to promote BAs excretion, reduce cholesterol synthesis and promote cholesterol consumption. AMPK, AMP-activated protein kinase; CYP7A1, cytochrome p-450 enzyme cholesterol 7α-hydroxylase; CYP27A1, cytochrome p-450 enzyme cholesterol 27α-hydroxylase; FXR, farnesoid X receptor; FGF 19, fibroblast growth factor 19; FGFR4, fibroblast growth factor receptor 4; GLP-1, glucagon-like peptide 1; GLP-1R, GLP-1 receptor; HMGCR, 3-hydroxy-3-methylglutaryl-CoA reductase; LPS, lipopolysaccharides; MAPK, mitogen-activated protein kinases; MDMs, microbiota-derived metabolites; Myd88, myeloid differentiation factor 88; PI3K-Akt, the phosphatidylinositol-3-kinase; PPAR, peroxisome proliferator-activated receptor γ; SBAs, secondary bile acid; SCAP, SREBP cleavage-activating protein; SCFAs, short-chain fatty acid; SHP, FXR-small heterodimer partner; SREBP, sterol regulatory element binding protein; TGR5, cell surface receptor G protein-coupled bile acid receptor 5; TLR4, Toll-like receptor 4; TMA, trimethylamine; TMAO, trimethylamine-N-oxide.

Statins can suppress TLR4 expression on cell membranes, including hepatocytes and enterocytes, modulate the TLR4-Myd88 signaling pathway, and prevent disease progression (134, 135). The TLR4-Myd88 pathway is restricted by SCFAs, and statins have been previously found to increase the SCFA-producing bacteria. Oral administration of butyrate reduces TLR4 and Myd88 expression in the mouse liver and repairs the damaged intestinal barrier (136). In another animal study, serum LPS levels were significantly decreased, and TLR4 expression in the liver and adipose tissue was significantly decreased after butyrate administration (137). SCFAs reduced the TLR4-Myd88 pathway activation by reducing LPS levels; however, whether SCFAs can directly affect the expression of TLR4 and Myd88 remains unclear. We summarize the effects of statins administration on cholesterol metabolism via gut microbiota and MDM that include SCFAs, BAs, TMAO, and LPS (Figure 2).

## Conclusion

This review aims to describe the effect of statins on serum LDL-C levels *via* the gut microbiota and to summarize the underlying mechanisms. Therefore, we focused on evidence describing statin alterations in gut microbiota abundance and their effects on gut MDM based on laboratory models and human data. As most studies have demonstrated, the LDL-C lowering effect of statins as direct inhibitors of HMGCR is potentially affected by the gut microbiota. In conclusion, scientific studies based on laboratory models have revealed that statins can alter the pathways associated with gut MDM, which in turn regulate the cholesterol metabolism and LDL-C levels. In particular, statins can modulate the abundance of gut microbiota producing SCFAs, SBAs, TMAO, and LPS, which in turn affect the AMPK-SREBP, FXR, PXR, FFAR2, and TLR4-Myd88 signaling pathways. Unfortunately, clinical trails on the effect of statins on serum LDL-C levels via the gut-liver axis are limited. However, preclinical studies have shown positive roles for the effects of statins on high LDL-C levels *via* the MDMs and gut liver axis, and it is fair to assume similar effects on the human gut and liver. Further clinical trials and experimental studies are required to better elucidate whether statins can effectively lower LDL-C levels through the gut microbiota.

## Author contributions

CS, MW, and LL designed and directed the manuscript. CS and ZW wrote the manuscript. LH and XZ revised the manuscript. JC searched the literature. ZY designed the illustrations and tables. All the authors approved the manuscript for publication, read, and approved the final manuscript.

## Funding

This work was supported by the National Natural Science Foundation of China (Nos. 81973689 and 82074254) and the Natural Science Foundation of Beijing Municipality (No. 7202176).

## Conflict of interest

The authors declare that the research was conducted in the absence of any commercial or financial relationships that could be construed as a potential conflict of interest.

## Publisher's note

All claims expressed in this article are solely those of the authors and do not necessarily represent those of their affiliated organizations, or those of the publisher, the editors and the reviewers. Any product that may be evaluated in this article, or claim that may be made by its manufacturer, is not guaranteed or endorsed by the publisher.
